# Citizen science in Lebanon—a case study for groundwater quality monitoring

**DOI:** 10.1098/rsos.181871

**Published:** 2019-02-27

**Authors:** Rima Baalbaki, Serine Haidar Ahmad, Wassim Kays, Salma N. Talhouk, Najat A. Saliba, Mahmoud Al-Hindi

**Affiliations:** 1 Department of Chemistry, Faculty of Arts and Sciences, American University of Beirut, Beirut, Lebanon; 2 Department of Chemical Engineering, Faculty of Engineering and Architecture, American University of Beirut, Beirut, Lebanon; 3 Nature Conservation Center, American University of Beirut, Beirut, Lebanon; 4 Department of Landscape and Ecosystem Management, Faculty of Agriculture and Food Science, American University of Beirut, Beirut, Lebanon

**Keywords:** citizen science, water quality, total coliforms, community-based monitoring

## Abstract

Over the past decade, several citizen science projects have been launched, with a smaller subset addressing citizen scientists' involvement in water quality monitoring. Most of these projects were conducted in developed countries and focused on qualitative assessment and measurements of a limited number of water quality parameters. Moreover, data generated by citizen scientists were mainly for monitoring purposes and rarely resulted in remedial measures. In this work, a collaborative citizen science approach involving local citizens and university researchers was applied to assess the groundwater quality in a Lebanese village. Using a mobile laboratory, winter and summer sampling campaigns were conducted and 12 physical, chemical and biological water quality parameters were tested. Results indicated that the data generated by the citizen scientists were comparable with those generated by university researchers for the majority of physical and chemical water quality parameters. However, the bacteriological test results showed a marked difference and may be attributed to the complexity of the testing procedure and quality of testing material. The collaborative and participatory approach resulted in building local capacity and knowledge and in the formation of a locally elected water committee which will be responsible for continuous monitoring of the groundwater resources.

## 1. Introduction

The concept of citizen science may have started in the early twentieth century when large amounts of data were generated for the National Audubon Society's Christmas Bird Count [1]. Over the last two decades, citizen science has seen a sharp rise in popularity in several fields. Recent projects include the monitoring of marine litter on coastal areas [2,3], identification of endangered bird and frog species [4], measuring levels of environmental exposure following oil spills [5] and evaluating the occurrence of Fibonacci spirals of sunflowers [6].

Fewer studies have shown the involvement of citizens in the monitoring of water resources [7–9]. Nevertheless, there are several citizen-based water resource monitoring programmes that have been initiated over the last couple of decades. The literature shows that there were/are several objectives for initiating these programmes and these include to monitor water quality and identify environmental problems [10–14] to initiate social change and/or legislation [15–17], and to provide environmental education and awareness [18–22].

Datasets generated by citizen science programmes were used to initiate crucial management actions against surface water quality deterioration by eutrophication and climate change or to show surface water quality improvement arising from advanced treatment or reduced run-off. Measurements of water clarity (Secchi disc) were recorded across eight states in the USA over a 74-year period [23]. Another study reported 23 years of temperature and water transparency (Secchi test) values by the lay community in Lake George, NY, to characterize long-term trends linking water clarity to climatic, chemical and recreational parameters [11]. A long-term citizen science project spanning a 22-year period of summertime water quality monitoring by volunteers (temperature, pH, salinity and dissolved oxygen), from 122 stations in 17 embayments in Buzzards Bay, Massachusetts, followed the effect of climate change on water eutrophication [14]. In France, 18 years' worth of citizen-generated data on river nutrients were analysed to determine whether nutrient concentrations were reduced during the intervening period [10]. Other citizen science projects investigated the impact of universities and professors in terms of their ability to establish a plan for an environmental monitoring organization and for educational purposes. These programmes targeted school children, undergraduates and graduates, as well as environmental group representatives. They aimed at engaging citizens in improving environmental conditions through connecting policy, science and action. Savan *et al*. [22] monitored biological ecosystem health parameters, mainly benthic invertebrates, lichen and *Escherichia coli* from 1997 to 2001; chemical indicator monitoring kits were expensive and thus not used; however, participants were taught about chemical water quality parameters and how these were affected by pollutants [22]. Sharpe & Conrad [24] emphasized the gap between ‘monitoring’ and ‘management’, i.e. when scientific data are collected but little is done concerning the optimization of the watersheds' management. The study addressed the case of Canadian province Nova Scotia, where community groups managed to collect water data starting in 1990, getting samples from more than 200 sites and 10 watersheds [24]. Water management concerns also engaged citizens and lay community in different educational programmes in Nevada [25], Michigan [26] and South Africa [21].

Table 1 summarizes citizen science water quality studies that have appeared in the literature over the past 20 years where it can be seen that the vast majority of studies were conducted in North America and Europe and where the quality of surface (lakes, rivers, and estuaries) and groundwater were investigated. Most of these studies measure more than one water quality parameter but, with one or two exceptions, are limited to no more than six parameters, which are usually a combination of physical, chemical and biological tests. It can also be seen from table 1 that for the vast majority of studies, the citizen scientists’ role in water quality projects was restricted to monitoring and data collection; the experts, whether scientists, university researchers or qualified laboratories undertook the water quality testing. In a small number of more recent studies, however, the role of the citizen scientists was expanded to include independent and simultaneous water quality testing, albeit for a very small number of water quality parameters. This approach, if properly implemented, will serve to empower community citizens with the skills needed to monitor their water quality long-term. It is also worth noting that a relatively small number of water quality projects were designed to include follow-up mitigation efforts, as the vast majority of these studies were intended for characterization and monitoring purposes. This issue is particularly relevant in the developing world where, in contrast to the developed world, where mitigation steps are usually implemented and monitored by regulated private/public sector entities, mitigation of documented water quality deterioration is rarely, if ever, implemented.

**Table 1.** Summary of citizen science projects related to water quality measurement.

reference	location	water body	citizen scientists’ role	water quality parameters measured
Obrecht *et al*. [13]	USA (Missouri)	lakes	sample collection	total phosphorus, total nitrogen, water transparency (Secchi disc), chlorophyll, temperature and total suspended solids
Au *et al*. [15]	Canada	creek	sample collection	coliforms, *E. coli*, phosphate, ammonia, pH, dissolved oxygen, hardness
Canfield *et al*. [27]	USA (Florida)	lake	sample collection	total phosphorus, total nitrogen, water transparency (Secchi disc), chlorophyll, pH, alkalinity, electrical conductivity
Nicholson *et al*. [12]	Australia	river	sample collection	turbidity, electrical conductivity, pH, total phosphorus
Savan *et al*. [22]	Canada	lakes and streams	sample collection	benthic invertebrates, lichen, *E. coli*
Boylen *et al*. [11]	USA (New York)	lakes and ponds	sample collection	water transparency (Secchi disc), temperature
Sharpe & Conrad [24]	Canada	lakes, streams, rivers, estuaries	sample collection	temperature, total suspended solids, faecal coliforms, dissolved oxygen
Roa Garcia & Brown [28]	Colombia	stream, headwater and household faucets	supervised testing	electrical conductivity, pH, total dissolved solids, dissolved oxygen, temperature, nitrate, phosphate, hardness, faecal and total coliforms
Loperfido *et al*. [29]	USA (Iowa)	river	supervised testing	nitrate, nitrite, total phosphorus, temperature, transparency
Stepenuck *et al*. [30]	USA (6 states)	recreational	independent testing	*E. coli*
Hoyer *et al*. [31]	USA (Florida)	lake	supervised testing	total phosphorus, total nitrogen and chlorophyll
Peckenham *et al*. [32]	USA (Maine)	groundwater	independent testing	electrical conductivity, chloride, nitrate, pH, hardness
Burgos *et al*. [16]	Mexico	river	sample collection	temperature, pH, hardness, alkalinity, turbidity, *E. coli*, coliforms
Latimore & Steen [26]	USA (Michigan)	lakes, streams	sample collection	water transparency (Secchi disc), total phosphorus, dissolved oxygen, temperature
Rheuban *et al*. [14]	USA (Massachusetts)	coastal water (estuary)	sample collection	temperature, pH, salinity, dissolved oxygen
Storey *et al*. [33]	New Zealand	streams	independent testing	water transparency, electrical conductivity, nitrates, dissolved oxygen, *E. coli*, temperature
Farnham *et al*. [34]	USA (NYC)	waterways	sample collection	faecal indicator bacteria
Jollymore at al. [35]	Canada	streams and rivers	independent testing	total suspended solids, nitrate, dissolved organic carbon
McGoff *et al*. [36]	UK	ponds, lakes and rivers	independent testing	nitrate, phosphate
Scott and Frost [37]	Canada	storm water pond	independent testing	turbidity, phosphate, nitrate
Thornhill *et al*. [17]	China	streams	supervised testing	turbidity, phosphate, nitrate
Abbott *et al*. [10]	France	river estuary	sample collection	phosphate, nitrate
Brouwer *et al*. [18]	Holland	tap water	independent testing	presence of bacteria
Wilson *et al*. [38]	USA (Alaska)	river	sample collection	pH, dissolved oxygen, conductivity, temperature, dissolved organic carbon, dissolved greenhouse gases, major ions, nutrients, trace metals, stable water isotopes

Lebanon suffers from high levels of water pollution, depletion of water resources and limited wastewater infrastructure and treatment facilities [39]. Most urban centres and villages in Lebanon depend on septic tanks or sewers with direct discharge into the environment [40,41]. High counts of faecal, total coliforms and diverse pollutants were reported [42,43] leading to the pollution of surface water and groundwater [44]. Lebanon also suffers from poor water management and governance practices and effluent streams data remain insufficient and poorly analysed [39]. Furthermore, water resource policy and planning are restricted by scarce, often unreliable data [45] and the absence of well-maintained and operated monitoring networks [9].

The specific objectives of this work were to (i) assess the interest of *citizens* in conducting water quality measurements, (ii) instigate a citizen-centric monitoring campaign to determine the quality of groundwater, (iii) compare the values obtained by citizen scientists and experts to assess the accuracy of the results, and (iv) analyse whether this participatory approach will lead to concrete actions in water resource management. This case study, to the authors' best knowledge, is the first of its kind in the Middle East and one of the very few from the developing world where the citizen science approach is used to gather water quality data from groundwater sources in a medium-sized village.

## 2. Material and methods

### 2.1. Study area

The selected area for this study is a village located in South Lebanon that occupies an area of approximately 12 km^2^ at an elevation of 400 m above sea level. No recent population census has been conducted but the population is estimated to be around 40 000. The village does not have a sewer network and wastewater is discharged to cesspools and septic tanks. As a result, septic tank infiltration may pose a serious problem on groundwater supplies given the increase in population and the scarcity in water supplies. Public water supply in the village is regulated by South Lebanon Water Establishment which distributes water from three artesian wells. Recently, one of these wells has been closed by the Ministry of Health after samples tested positive for bacterial contamination. To compensate for the water shortage, residents have resorted to privately owned wells and several well owners have been selling water from their private wells.

Lebanon has a Mediterranean climate characterized by rainy winters and hot dry summers. Most, if not all, of the precipitation falls during the cold season (between December and March) with an average daily high temperature below 18°C. This leaves the warm season (between June and October) with dry and humid weather with an average daily high temperature above 28°C.

### 2.2. Citizen participation

#### 2.2.1. Research ethics approval

The research proposal, oral informed consent documentation, presentations, data collection forms, questionnaires and surveys related to this project were reviewed by the American University of Beirut (AUB) Institutional Review Board (IRB). In addition, all researchers involved in this study took the Social & Behavioral Research—Basic Course which is a Web-based course offered by the Collaborative Institutions Training Initiatives (CITI) Programme to provide research ethics education to the research community.

#### 2.2.2. Citizens’ involvement

The framework adopted for citizen involvement is outlined in Talhouk *et al*. [46]. In 2014, the Nature Conservation Center (NCC), of the American University of Beirut, established a community outreach programme where participatory mapping was conducted by a university team with stakeholders in 50 villages to identify local natural and cultural landmarks. One of the outcomes of this work was the identification of clean water as a top environmental priority for one of the villages in the study group. Representatives from this village were contacted by the university team in early 2015 to verify interest in the project. In view of the university team's previous knowledge, and after the identification of various stakeholders (municipality, well owners, non-governmental organizations, community centres, etc.), meetings were arranged between representatives of the research team and the stakeholders. The village local community was invited through the municipality to attend a project introductory presentation on 31 October 2015. In this presentation, the project objectives and proposed analysis methods were outlined.

After the presentation, a call for participation was opened and citizens showing interest in participation were asked to complete a perception questionnaire about water quality. The water perception questionnaire answered by the citizens on the day of project introduction was made up of eight questions that examined their knowledge concerning household water at their residence, the health implications of using polluted water and the general sources of water pollution. Participation was open to all; however, the vast majority of volunteers were women. This gender imbalance may be in response to the composition of the university team which consisted mostly of women scientists. Another possibility may be that women leading a local women club took the initiative to recruit participants. The university team did not influence participation and hence did not enforce gender balance among the citizen scientist volunteers.

Several meetings and workshops were arranged with the participating/volunteer citizens. These meetings/workshops were used to explain various aspects of water quality (standards, parameters, significance of these parameters, testing procedures) and the participating citizens were trained to perform the various water quality tests. The presentation and workshops were planned to accommodate local interpretive capabilities of citizen scientists; fact sheets and infographics were prepared in Arabic for each water quality parameter and included background information, illustrated methodology and safety information.

### 2.3. Sampling and water quality testing

**Figure 1.**
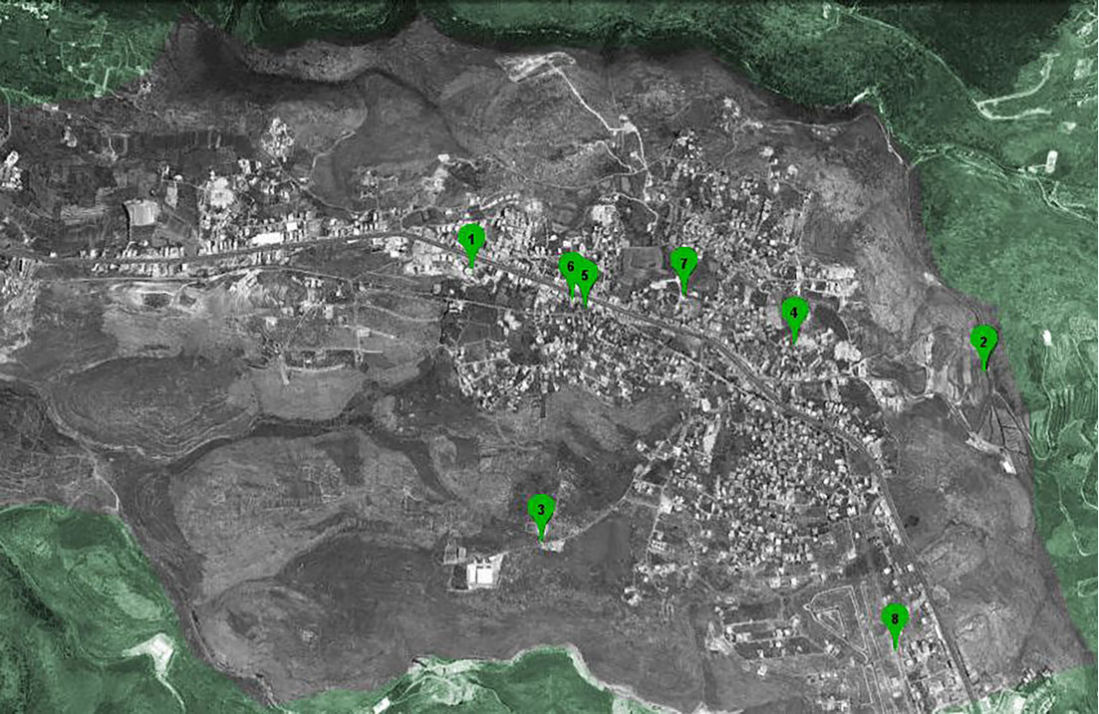
A Google Earth aerial map of the sampling site.

The location of the water sources was determined using a collaborative effort between representatives from the university, the local municipality, the water authority, private well owners and volunteers (figure 1). Water samples were taken from three public wells, three private wells and two water storage tanks. The number of collected samples from each water source is summarized in electronic supplementary material, table S1. Sampling was conducted by both trained citizens and researchers following the recommendations of the US EPA (United States Environmental Protection Agency) [47].

Prior to every sampling campaign, university researchers would contact the local village representative to arrange a mutually convenient time for the citizen scientists and university team to meet. The village representative would then contact the well owners and the municipality to advise them that sampling will take place at the agreed-upon time. The pre-sampling meetings were held at the local municipality and a team consisting of two university researchers and several citizen scientists (the numbers varied but there were a minimum of three and a maximum of five) would set out together to collect the samples from each of the locations shown in figure 1. During the winter campaigns, the collected and labelled samples were stored in the municipality's dispensary overnight; the analysis was then conducted the following day. On the other hand, during the summer campaigns, the collection and analysis took place during the same day.

Water samples were tested for a total of nine physical and chemical (pH, electrical conductivity, turbidity, hardness, alkalinity, nitrates, nitrites, ammonia and phosphates) and three biological parameters (total coliforms, faecal coliforms and *E. coli*). For this purpose, simple field testing kits and laboratory supplies were purchased or assembled by the team. The selection criteria for the operational methods of the kits included ease of use, short testing duration and moderate sensitivity. A summary of the tested water quality parameters, designated instruments or kits used and method is presented in table 2.

**Table 2.** Summary of the water quality parameters, instrument used and testing method.

parameters	instrument/test kit	method
physical		
PH	Hach HQ40D-IntelliCALTM PHC101	electrode method
conductivity	Hach HQ40D-IntelliCALTM CDC401	electrode method
turbidity	Eutech TN100	nephelometry
chemical		
alkalinity	Hach AL-AP Test kit	drop count titration/sulfuric acid
hardness	Hach HA-71A Test kit	drop count titration/EDTA
nitrates	HACH DR900 Colorimeter/Hach NitraVer 5 Reagent Powder Pillows	cadmium reduction method
nitrites	HACH DR900 Colorimeter/Hach NitriVer 3 Reagent Powder Pillows	diazotization method
phosphorus	HACH DR900 Colorimeter/Hach PhosVer 3 Reagent Powder Pillows	ascorbic acid method
ammonia	HACH DR900 Colorimeter/Hach Ammonia Reagent set	salicylate method
biological		
total coliforms	Hach m-ColiBlue24 Broth & Endo nutrient pad sets	membrane filtration method
faecal coliforms	m FC broth & m FC nutrient pad sets	membrane filtration method

Conductivity, pH and dissolved oxygen were measured by submerging the respective electrode of the Hach HQ40D meter in the water sample after rinsing it with deionized water. For turbidity measurements, the sample vial was rinsed with the sample three times, and then it was filled to line mark, wiped with a lint-free cloth and placed in the meter to be measured. All electrodes of the Hach HQ40D and the turbidity meter were calibrated prior to use. Alkalinity was measured using drop count titration by sulfuric acid after the addition of phenolphthalein and Bromcresol Green-Methyl Red indicators, respectively. Water total hardness was also measured using drop count titration by EDTA (Ethylene-diaminetetraacetic acid) after the addition of buffer and Calmagit indicator. Nitrogen-nitrate (NO_3_-N), nitrogen-nitrite (NO_2_-N), nitrogen-ammonia (NH_3_-N) and orthophosphate were measured by colorimetric assays using the Hach DR900 handheld colorimeter. For comparison purposes with the water quality guidelines, NO_3_-N, NO_2_-N and NH_3_-N were converted to $NO_{3}^{-}$ , $NO_{2}^{-}$ and $NH_{4}^{+}$ by multiplying each value by 4.4, 3.3 and 1.3, respectively.

For the summer and winter campaigns, all the physical and chemical water quality parameters were tested independently by both the citizens and laboratory technicians. However, bacteriological tests for winter samples were conducted by university technicians because the sterilizing filtration equipment was not available at the village. For the summer samples, bacteriological tests were performed by citizens using sterile disposable filtration units and nutrient pad sets.

## 3. Results

### 3.1. Community participation

After the project introductory presentation, 46 citizens were interested in participating in the project of which 65.2% were adult females and 34.8% were young school students (6th grade); however, this number dropped to a total of 26 citizens, predominantly women, who participated in the water quality testing over six sessions. The age of the participants varied between 16 and 62 and averaged 35 years old. Their educational level also varied between primary, secondary and tertiary education. It is worth noting that female participants were mostly interested in conducting water test analysis, while the male participants were more engaged in the sample collection. This observation may be attributed to the local culture where men usually work outdoors while women partake in indoor work activities.

The results of the questionnaire are summarized in electronic supplementary material, table S2. Interestingly only 16% of the citizens who answered the questionnaire relied on municipal water while the remaining citizens purchased water from local wells or water tankers. Forty-three per cent of the participants were not aware of the quality of their household water and whether it was fit for the intended purpose (drinking and cooking). Most of the respondents (43%) stated that the water at their household was not potable because it contained microbes (13%), it had a non-desirable taste (11%), or it was contaminated with chemicals (7%). Most of the participants (44%) did not answer the question related to potential sources of water pollution.

During the water sampling and testing campaigns, some new citizen scientists joined in the middle of the project while others left, but there was always a core group which persisted throughout (and beyond) the duration of the project and this proved crucial for peer training newcomers. Throughout the project, the university team served as a knowledge resource for the citizen scientists and responded to all water quality-related inquiries, whether they were directly related to the project or not.

The water test results, generated by the university team and the citizen scientists, were shared in a public seminar organized by the local authorities and attended by all stakeholders including citizen scientists, private well owners, representatives from the municipality, representatives from a local school and concerned residents. Recognition and pride in local resident participation were expressed by all attendees. Women scientists, now better informed about the water quality in the village voiced their opinion and concerns to the local authorities during the public session.

### 3.2. Groundwater quality

Table 3 lists the total average results for all nine physical and chemical water quality parameters recorded by the citizen scientists and university researchers. The test results were compared with the Lebanese water quality standard to identify non-conforming values. For five out of the nine parameters (pH, conductivity, turbidity, hardness and alkalinity), all of the test results for both the summer and winter campaigns were within the values recommended by the Lebanese standards. For nitrate, nitrite, ammonia and phosphate, the vast majority of the test results were within the recommended values; the exceptions were for well 7 where all four parameters exceeded the Lebanese standard values during two testing campaigns and for well 2 where the nitrite and phosphate levels exceeded the standard for one of the testing campaigns.

**Table 3.** Average results for the water quality parameters recorded by citizen scientists and university researchers.

parameter	*N*	citizen	university researchers
pH	39	7.71 ± 0.20	7.68 ± 0.30
conductivity (µS/cm)	39	550.6 ± 93.9	540.9 ± 93.8
turbidity (NTU)	33	1.86 ± 3.80	1.87 ± 5.20
hardness (mg l^−1^)	39	330 ± 57	315 ± 48
alkalinity (mg l^−1^ as CaCO_3_)	39	284 ± 59	284 ± 51
nitrates (mg l^−1^)	39	15.7 ± 12.9	15.1 ± 11.6
nitrites (mg l^−1^)	39	0.08 ± 0.30	0.09 ± 0.30
ammonia (mg l^−1^)	39	0.15 ± 0.10	0.11 ± 0.10
phosphates (mg l^−1^)	39	0.35 ± 0.30	0.52 ± 0.40

### 3.3. Comparison between citizens' data and expert personnel's data

A comparison between data generated by citizen scientists and those by university researchers for the nine physical and chemical water quality parameters was established based on paired *T*-test analysis for the dry/summer and wet/winter campaigns (these are summarized in electronic supplementary material, table S3). There was no significant difference between the citizen scientists' data and the university researchers’ data for seven of the nine water quality parameters: pH, conductivity, turbidity, alkalinity, nitrates, nitrites and ammonia. However, there were significant differences for the hardness and phosphate data (table 3). Table 4 lists the results recorded by the citizen scientists during the summer and winter campaigns.

**Table 4.** Citizen measurements (average ± s.d.) of nine water quality parameters at eight sites.

site	season	pH	conductivity (µS/cm)	turbidity (NTU)	hardness (mg l^−1^)	alkalinity (mg l^−1^ as CaCO_3_)	nitrates (mg l^−1^)	nitrites (mg l^−1^)	ammonia (mg l^−1^)	phosphates (mg l^−1^)
1	winter	7.31	633	19.97	393.3	280	14.17	0	0.13	0.44
	summer	7.4 ± 0.06	611 ± 62.23	5.54 ±	342 ± 72.55	300 ± 56.57	23.02 ± 3.76	0.01 ± 0.01	0.14 ± 0.01	0.48 ± 0.49
2	winter	8.03 ± 0.11	433 ± 18.01	0.25 ± 0.08	250.8 ± 54.97	206.7 ± 57.74	6.64 ± 1.17	0.01 ± 0.01	0.09 ± 0.02	0.24 ± 0.1
	summer	7.83 ± 0.14	420 ± 1.53	0.06 ± 0.08	250.8 ± 26.12	273.3 ± 70.24	4.43 ± 2.69	0.01 ± 0.01	0.33 ± 0.38	0.77 ± 0.76
3	winter	7.62 ± 0.32	586 ± 20.65	1.54 ± 1.27	393.3 ± 17.1	333.3 ± 23.09	8.71 ± 0.26	0.01 ± 0.02	0.1 ± 0	0.29 ± 0.15
	summer	7.66 ± 0.03	590 ± 1.53	0.14 ± 0.09	330.6 ± 54.97	333.3 ± 61.1	7.08 ± 1.93	0.03 ± 0.01	0.12 ± 0.01	0.13 ± 0.02
4	winter	7.96 ± 0.04	396 ± 27.58	0.28 ± 0.02	333.5 ± 84.64	220 ± 56.57	7.75 ± 0.31	0.01 ± 0	0.1 ± 0.01	0.42 ± 0.33
	summer	7.86 ± 0.07	482 ± 100.78	1.13 ± 1.57	273.6 ± 17.1	246.7 ± 50.33	5.02 ± 0.92	0.01 ± 0.01	0.11 ± 0.01	0.42 ± 0.34
5	winter	7.48 ± 0.13	543 ± 39.89	0.93 ± 0.59	330.6 ± 9.87	246.7 ± 57.74	20.07 ± 0.68	0.02 ± 0	0.12 ± 0.01	0.39 ± 0.28
	summer	7.4 ± 0.15	582 ± 6.36	0.14 ± 0.19	350.6 ± 12.09	350 ± 14.14	17.93 ± 11.58	0.02 ± 0	0.12 ± 0.02	0.37 ± 0.46
6	winter	7.93 ± 0.12	538 ± 42.77	0.45 ± 0.13	364.8 ± 54.97	293.3 ± 23.09	19.04 ± 2.34	0.02 ± 0.01	0.09 ± 0.01	0.17 ± 0.17
	summer	7.74 ± 0.11	584 ± 15.37	0.1 ± 0.14	324.9 ± 29.62	293.3 ± 92.38	22.58 ± 2.03	0.02 ± 0.01	0.12 ± 0.01	0.14 ± 0.05
7	winter	7.79 ± 0.27	727 ± 41.24	5.59 ± 3.76	381.9 ± 26.12	293.3 ± 50.33	51.21 ± 12.79	0.78 ± 0.64	0.34 ± 0.2	0.4 ± 0.31
8	winter	7.69 ± 0.1	553 ± 10.69	1.17 ± 0.38	353.4 ± 64.74	313.3 ± 30.55	8.41 ± 0.89	0.03 ± 0.01	0.1 ± 0.01	0.28 ± 0.08

Bacteriological test, represented by total coliforms, faecal coliforms and *E. coli*, performed by university researchers during the winter (wet/cold) campaign are presented in table 5. The biological test results for the summer campaign as recorded by citizens and university researchers are also shown in table 5. For the winter campaign, faecal coliforms were not found in any of the tested samples while *E. coli* was only found in one sample from site 1 and another from site 8, and, with the exception of sites 3 and 4 (which were devoid of all forms of bacterial contamination), all the other sites exhibited total coliforms contamination to varying levels and occurrence with sites 1 and 7 exhibiting the highest counts. On the other hand, during the summer campaign, total coliform counts were found in all six sites and faecal coliforms were found in sites that did not show any contamination during the winter campaign, namely sites 3 and 6. It can also be seen from table 5 that there are several discrepancies between citizen scientists' results and those recorded by the university researchers.

**Table 5.** Bacteriological test results during the winter and summer campaigns. Y: bacteria was detected; N: no bacteria was detected; –: no results.

site	winter				summer				
	date	Results generated by university researchers only			date	university researchers		citizen scientists	
		total coliforms (CFU/100 ml)	faecal coliforms (CFU/100)	*E. coli* (CFU/100 ml)		total coliforms	faecal coliforms	total coliforms	faecal coliforms
1	25 Nov 15	28	0	0	3 Aug 16	Y	N	Y	N
1	9 Dec 15	32	0	0	17 Aug 16	–	–	–	–
1	17 Feb 16	546	0	18	10 Sep 16	Y	N	Y	N
2	25 Nov 15	0	0	0	3 Aug 16	Y	N	Y	N
2	9 Dec 15	288	0	0	17 Aug 16	N	N	Y	Y
2	17 Feb 16	0	0	0	10 Sep 16	Y	N	Y	–
3	25 Nov 15	0	0	0	3 Aug 16	Y	Y	Y	N
3	9 Dec 15	0	0	0	17 Aug 16	Y	Y	Y	N
3	17 Feb 16	0	0	0	10 Sep 16	Y	Y	–	–
4	25 Nov 15	0	0	0	3 Aug 16	Y	N	Y	N
4	9 Dec 15	–	–	–	17 Aug 16	Y	N	Y	N
4	17 Feb 16	0	0	0	10 Sep 16	Y	N	Y	–
5	25 Nov 15	0	0	0	3 Aug 16	N	N	Y	N
5	9 Dec 15	8	0	0	17 Aug 16	Y	N	Y	N
5	17 Feb 16	0	0	0	10 Sep 16	–	–	–	–
6	25 Nov 15	48	0	0	3 Aug 16	Y	Y	Y	Y
6	9 Dec 15	84	0	0	17 Aug 16	Y	Y	Y	N
6	17 Feb 16	2	0	0	10 Sep 16	Y	Y	Y	–
7^a^	25 Nov 15	616	0	0	3 Aug 16	–	–	–	–
7^a^	9 Dec 15	580	0	0	17 Aug 16	–	–	–	–
7^a^	17 Feb 16	68	0	0	10 Sep 16	–	–	–	–
8^a^	25 Nov 15	84	0	0	3 Aug 16	–	–	–	–
8^a^	9 Dec 15	232	0	8	17 Aug 16	–	–	–	–
8^a^	17 Feb 16	12	0	0	10 Sep 16	–	–	–	–

^a^During the summer campaign, well 7 was shut off by the private owner and well 8's main outlet was directed to a swimming pool preventing water sampling activities.

## 4. Discussion

This study required assembling a mobile laboratory, developing and translating experimental protocols, preparing data forms and administering pre- and post-test presentations. These tools contributed to building local capacity, combining knowledge into actions, and ensuring the development of sustainable solutions. Although the initial objectives of the project were grounded in the citizen science framework, the project evolved to lead to (i) the formation of a local water committee whose mandate is to follow up on water testing at the village, (ii) the initiation of corrective measures such as the replacement of several interconnecting pipes for one of the contaminated storage tanks, and (iii) the use of low-cost disinfection treatment to the water wells that exhibited low level of bacterial contamination. The project, in terms of training, data collection and data analysis, occurred during the period January 2015 to December 2016. Remedial measures (piping modifications and disinfection equipment), initiated and procured by the local municipality in coordination with the water committee/university researchers were implemented in February 2017 and subsequent tests during March 2017 confirmed the effectiveness of these measures. The project was ‘officially’ concluded in May 2017. A ‘concluding meeting’ was held at the village on the 13 May 2017 where university researchers and the citizen scientists (as well as several members of the local municipality and water authority) were in attendance. Assurances were given during the meeting that water monitoring by the members of the water committee, and other volunteers, will continue and additional financial implications (i.e. new kits and consumables) will be factored into the local municipality's budget. It should be noted that the university team handed over a complete set of testing kits which would have been adequate for several testing campaigns. Follow-up by the university team over the period May 2017–May 2018 confirmed that partial, semi-regular water sampling and testing (bacteria, turbidity and hardness) was conducted at various locations and on a bi-monthly basis and that the quality of the water was consistent with previous tests. However, in a more recent follow-up discussion (early December 2018), members of the water committee confirmed that testing has not occurred for almost two months as a result of a dispute between the water committee and the municipality over the results of one of the sites. It appears that bacteriological contamination was found in one of the wells and the owner/municipality refused to accept the result and hence to proceed with remedial action. In addition, the water committee members intimated that future testing may be in jeopardy due to unavailability of testing kits (budgetary constraints have hindered the procurement process). It is anticipated that these set-backs will be resolved in due course as members of the committee have stated that both parties have agreed, in principle, to resolve this issue by seeking the opinion of the water authority.

The key components that ensured the success of the project are the long-time trust between the community and the university (American University of Beirut-Nature Conservation Center), the flexibility in setting the university-staff visits according to the local residents' schedule, and the respect of the local culture and community dynamics. Research and community development in the context of an academic project is usually a high priority to the academic investigators. However, the same situation does not apply to citizens who are engaged in the project as volunteers. This creates an imbalance in the work progress because both parties are equally involved in the project. As such, citizen science projects conducted in a public and private participatory approach puts the conventional methodology that is usually bound to timeline and clear deliverables into question. Moreover, the university was keen on building the trust of the community by sharing the non-complying results with the stakeholders and discussing with them possible remedial solutions before involving the public at large. All findings were disseminated in public seminars, which served as a platform for discussion among citizens, the local authority and the academic experts. One additional outcome of the study is the development of a roadmap for testing the water in the village or at home, to identify possible quick remedial solutions and to establish links with the Water Authority personnel. Contrary to other studies where citizens were mainly involved as sample [48] or data [9] collectors, this project allowed citizens to play a major role in analysing the data, discussing corrective measures and ensuring sustainable solutions.

Lebanese municipal water, often sourced from groundwater, is generally perceived to be unsafe to drink, primarily because of contamination in pipelines or in local storage tanks. Most Lebanese households use bottled water for drinking purposes. However, municipal water is continuously monitored by governmental authorities and should in principle comply with the Lebanese drinking water standard. Results show that for a number of wells, the levels of nitrate, nitrite and phosphates exceeded the Lebanese standard and that the presence of faecal coliforms was also observed in a number of locations. The elevated chemical levels and the bacteriological contamination may have resulted from agricultural water run-off and/or septic tank infiltration into the local groundwater. It is important to note that the chemical and bacteriological findings confirm the general perception of local citizens that were assessed during the preliminary stage of the project. The results of this work also highlight the ability of citizens to produce reliable and repeatable data for a substantial number of water quality indicators. As reported earlier, the citizen scientists’ results and those generated by the university researchers for seven out of the nine physical and chemical parameters, namely conductivity, pH, turbidity, alkalinity, nitrate, nitrite and ammonia, were in good agreement. Two out of the nine physical/chemical tests, namely hardness and phosphate, and all three bacteriological tests showed discrepancies between the citizens and the university researchers. This could be ascribed to the difficulty in discerning the change of the colour indicator in the hardness test, the sensitivity of the experiment in the case of phosphate and the complexity of the bacteriological tests. Agreement between data produced by citizen scientists and university researchers for most of the physical and chemical parameters considered in this work has been reported by several authors [27,32,33,35,37]. However, for the case of the phosphate and bacteriological tests, the literature is rather contradictory where some workers have reported agreement [31,36] and others have reported divergence [13,29] between the datasets. Other citizen ‘biases’ emanating from previous spatial or temporal events [9,35,49] do not apply to this study because citizen scientists were conducting the tests on coded water samples that were doubly blinded.

In summary, and despite some challenges faced throughout the project duration and beyond, the evolved methodology/framework of this work involved several sequential and iterative tasks which were performed either separately or synergistically by the university team and the citizen scientists. The key tasks may be summarized chronologically: those performed during the conception stage, those performed during the project implementation stage and those performed (and are still being performed) after the end date of the project. During the ‘conception stage’, the following activities were completed: (i) formation of university ‘outreach team’ to coordinate and maintain strong and long-term relations with the local community, (ii) site visits to reach consensus with the local stakeholders on an action/implementation plan, (iii) formation of a local ‘citizen scientists committee’ to implement the project, and (iv) selection of chemical, physical and bacteriological tests to ensure ease of use and reproducibility. During the ‘implementation stage’, the following tasks were performed: (i) finalization of the sampling locations, (ii) training workshops for the ‘citizen science committee’ on water quality testing (these were conducted prior to the actual sampling and testing), (iii) simultaneous sampling and testing by the university team and the citizen scientists, (iv) repeating the tests, using the same samples, at the university's certified laboratory for verification purposes, and (v) open discussion between the university team, the local citizen committee and all other stakeholders regarding the water testing results. After the project completion and in order to ensure sustainability beyond the end date, several actions were implemented including stakeholder meetings to discuss short- and long-term water treatment solutions and the formation of a ‘water committee’ to continue the water quality testing at the village level and to follow up the implementation of short/long-term treatment solutions.

## 5. Conclusion

In this work, a citizen science approach was undertaken to determine the quality of commonly used water sources in a Lebanese village. Twelve physical, chemical and bacteriological water quality indicators were tested. The results showed no statistical differences in 7 out of the 11 tests. Trained citizens showed that they are as capable as university researchers in providing reliable, repeatable and accurate physical and chemical water quality test data. Discrepancies were attributed to the sensitivity and the complexity of certain chemical and biological procedures. Test results indicated that agricultural run-off and the preponderance of septic tanks in the area combined with the absence of wastewater treatment may have inadvertently resulted in groundwater pollution. The results were shared with the citizens in presentations followed by active discussions that focused mainly on corrective measures. Ultimately, the success of the project was primarily due to the collaborative efforts put forth by the community and the university stakeholders. Major outcomes included the formation of a water committee at the village, the development of a roadmap that allows citizens to either conduct their own water tests or report to the public authority, and the establishment of a local laboratory at the municipality that will ensure sustainability of water quality monitoring.

## Supplementary Material

Supplementary Tables

## Supplementary Material

Citizen Science Data 2

## Permission to carry out fieldwork

No permissions and licenses were required prior to conducting our research.

## Data accessibility

All data used in this paper have been uploaded as part of the electronic supplementary material.
