# Supplementary material for: Citizen science in Lebanon—a case study for groundwater quality monitoring
Source: R Soc Open Sci. 2019 Feb 27;6(2):181871. doi: 10.1098/rsos.181871 (PMC6408373; doi:10.1098/rsos.181871)
Supplement: Supplementary Tables [file rsos181871supp1.docx]

Supplementary Tables

Table S1. Sampling dates

|  |  |  | **Winter** |  |  | **Summer** |  |
| --- | --- | --- | --- | --- | --- | --- | --- |
| Site | Type | Nov 25, 2015 | Dec 9, 2015 | Feb 17, 2016 | Aug 3, 2016 | Aug 17, 2016 | Sep 10, 2016 |
| 1 | Public | ✓ | ✓ | ✓ | ✓ | ✓ | ✓ |
| 2 | Public | ✓ | ✓ | ✓ | ✓ | ✓ | ✓ |
| 3 | Public | ✓ | ✓ | ✓ | ✓ | ✓ | ✓ |
| 4 | Storage Tank | ✓ |  | ✓ | ✓ | ✓ | ✓ |
| 5 | Private | ✓ | ✓ | ✓ | ✓ | ✓ |  |
| 6 | Storage Tank | ✓ | ✓ | ✓ | ✓ | ✓ | ✓ |
| 7 | Private | ✓ | ✓ | ✓ |  |  |  |
| 8 | Private | ✓ | ✓ | ✓ |  |  |  |

Table S2. Water perception questionnaire results

| **Questions** | **Answers** | **Number of responses** |
| --- | --- | --- |
| What is the source of water used in your house? | municipality water | 7 |
|  | purchased water | 5 |
|  | well | 5 |
|  | river or stream | 1 |
|  | municipality and purchased water | 9 |
|  | municipality and well | 3 |
|  | purchased water and well | 2 |
|  | no answer | 13 |
| Do you treat water before using it? | yes | 4 |
|  | no | 29 |
|  | no answer | 13 |
| How do you treat water? | using a filter | 4 |
|  | using chlorine | 1 |
|  | no answer | 41 |
| Do you think that this water is of good quality? | no | 5 |
|  | medium quality | 6 |
|  | yes | 2 |
|  | I don't know | 20 |
|  | no answer | 13 |
| Do you think this water is potable? | yes | 1 |
|  | no | 20 |
|  | I don’t know | 8 |
|  | no answer | 17 |
| If you answered the previous question by no, why? | because it tastes weird or is salty | 5 |
|  | because it is dirty | 2 |
|  | because it is contaminated with microbes | 6 |
|  | because it is contaminated with chemicals | 3 |
|  | because it tastes weird or is salty and because of its color | 1 |
|  | because it tastes weird, is dirty and contaminated with microbes and chemicals | 1 |
|  | because it is dirty and contaminated with microbes and chemicals | 1 |
|  | no answer | 27 |
| In your opinion, what is the health effect of using polluted water? | It is not harmful | 6 |
|  | it causes diarrhea | 2 |
|  | it causes inflammations like hepatitis and cholera | 11 |
|  | has other effects | 3 |
|  | It causes diarrhea and inflammations like hepatitis and cholera | 5 |
|  | It causes diarrhea and inflammations like hepatitis and cholera and has other effects | 2 |
|  | no answer | 17 |
| What is the cause of water pollution | contaminated with sewage water | 6 |
|  | I don't know | 9 |
|  | contaminated with sewage water, fertilizers and pesticides | 3 |
|  | contaminated with sewage water and airborne pollutants | 1 |
|  | contaminated with fertilizers, pesticides and airborne pollutants | 1 |
|  | contaminated with sewage water, fertilizers, pesticides, airborne pollutants and garbage | 2 |
|  | contaminated with sewage water, fertilizers, pesticides and garbage | 3 |
|  | no answer | 20 |

Table S3 T-test analysis for the dry/summer and wet/winter campaigns

| **Paired Samples Test** | | | | | | | | | |
| --- | --- | --- | --- | --- | --- | --- | --- | --- | --- |
|  | | Paired Differences | | | | | t | df | Sig. (2-tailed) |
|  |  | Mean | Std. Deviation | Std. Error Mean | 95% Confidence Interval of the Difference | |  |  |  |
|  |  |  |  |  | Lower | Upper |  |  |  |
| Pair 1 | PH_Lab - PH_Village | -0.02615 | 0.16497 | 0.02642 | -0.07963 | 0.02732 | -0.990 | 38 | 0.328 |
| Pair 2 | Cond_Lab - Cond_Village | -9.692 | 30.576 | 4.896 | -19.604 | 0.219 | -1.980 | 38 | 0.055 |
| Pair 3 | Turbidity_Lab - Turbidity_Village | 0.00758 | 1.85601 | 0.32309 | -0.65054 | 0.66569 | 0.023 | 32 | 0.981 |
| Pair 4 | Hardness_Lab - Hardness_Village | -15.3462 | 42.0320 | 6.7305 | -28.9714 | -1.7209 | -2.280 | 38 | 0.028 |
| Pair 5 | Alkalinity_Lab - Alkalinity_Village | 0.513 | 59.558 | 9.537 | -18.793 | 19.819 | 0.054 | 38 | 0.957 |
| Pair 6 | Nitrates_Lab - Nitrates_Village | -0.612897 | 6.616047 | 1.059415 | -2.757572 | 1.531777 | -0.579 | 38 | 0.566 |
| Pair 7 | Nitrites_Lab - Nitrites_Village | 0.010385 | 0.040341 | 0.006460 | -0.002692 | 0.023462 | 1.608 | 38 | 0.116 |
| Pair 8 | Ammonia_Lab - Ammonia_Village | -0.032897 | 0.106505 | 0.017055 | -0.067423 | 0.001628 | -1.929 | 38 | 0.061 |
| Pair 9 | Phospahtes_Lab - Phospahtes_Village | 0.171026 | 0.514044 | 0.082313 | 0.004392 | 0.337659 | 2.078 | 38 | 0.045 |
